# Potassium *N*,4-dichloro­benzene­sulfonamidate monohydrate

**DOI:** 10.1107/S1600536811020319

**Published:** 2011-06-04

**Authors:** B. Thimme Gowda, Sabine Foro, K. Shakuntala

**Affiliations:** a Department of Chemistry, Mangalore University, Mangalagangotri 574 199, Mangalore, India; b Institute of Materials Science, Darmstadt University of Technology, Petersenstrasse 23, D-64287 Darmstadt, Germany

## Abstract

The structure of the title salt hydrate, K^+^·C_6_H_4_Cl_2_NO_2_S^−^·H_2_O, shows each of the sulfonyl O and water O atoms to be bidentate bridging. The hepta­coordinated K^+^ cation is connected to two water O atoms, four sulfonyl O atoms and one Cl atom. The crystal structure comprises sheets in the *bc* plane which are further stabilized by O—H⋯N hydrogen bonds.

## Related literature

For our studies into the effect of substituents on the structures of *N*-haloaryl­sulfonamides, see: Gowda *et al.* (2007**a* ▶,b* ▶) and on the oxidative strengths of *N*-halolaryl­sulfonamides, see: Gowda & Shetty (2004 ▶); Usha & Gowda (2006 ▶). For similar structures, see: George *et al.* (2000 ▶); Olmstead & Power (1986 ▶). For the preparation of the title compound, see: Jyothi & Gowda (2004 ▶).
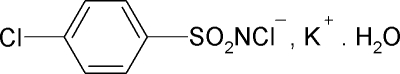


## Experimental

### 

#### Crystal data

K^+^·C_6_H_4_Cl_2_NO_2_S·H_2_O
*M* *_r_* = 282.18
Monoclinic, 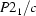

*a* = 15.487 (1) Å
*b* = 10.0620 (8) Å
*c* = 6.8061 (5) Å
β = 99.888 (7)°
*V* = 1044.84 (13) Å^3^
*Z* = 4
Mo *K*α radiation
μ = 1.20 mm^−1^
*T* = 293 K
0.42 × 0.42 × 0.30 mm

#### Data collection

Oxford Diffraction Xcalibur diffractometer with a Sapphire CCD detector
Absorption correction: multi-scan (*CrysAlis RED*; Oxford Diffraction, 2009 ▶) *T*_min_ = 0.633, *T*_max_ = 0.715
3796 measured reflections
2139 independent reflections
1962 reflections with *I* > 2σ(*I*)
*R*_int_ = 0.009

#### Refinement

*R*[*F* ^2^ > 2σ(*F* ^2^)] = 0.024
*wR*(*F* ^2^) = 0.067
*S* = 1.04
2139 reflections
134 parameters
2 restraints
H atoms treated by a mixture of independent and constrained refinement
Δρ_max_ = 0.39 e Å^−3^
Δρ_min_ = −0.28 e Å^−3^

### 

Data collection: *CrysAlis CCD* (Oxford Diffraction, 2009 ▶); cell refinement: *CrysAlis RED* (Oxford Diffraction, 2009 ▶); data reduction: *CrysAlis RED*; program(s) used to solve structure: *SHELXS97* (Sheldrick, 2008 ▶); program(s) used to refine structure: *SHELXL97* (Sheldrick, 2008 ▶); molecular graphics: *PLATON* (Spek, 2009 ▶); software used to prepare material for publication: *SHELXL97*.

## Supplementary Material

Crystal structure: contains datablock(s) I, global. DOI: 10.1107/S1600536811020319/tk2749sup1.cif

Structure factors: contains datablock(s) I. DOI: 10.1107/S1600536811020319/tk2749Isup2.hkl

Supplementary material file. DOI: 10.1107/S1600536811020319/tk2749Isup3.cml

Additional supplementary materials:  crystallographic information; 3D view; checkCIF report

## Figures and Tables

**Table 1:** Selected bond lengths (Å)

K1—O2	2.8576 (13)
K1—O2^i^	2.6940 (13)
K1—O1^ii^	2.7965 (13)
K1—O1^iii^	2.8547 (13)
K1—O3^iv^	2.8714 (15)
K1—O3^v^	3.1905 (16)
K1—Cl2	3.3944 (6)

Symmetry codes: (i) 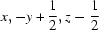; (ii) 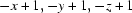; (iii) 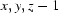; (iv) 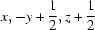; (v) 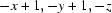.

**Table 2:** Hydrogen-bond geometry (Å, °)

*D*—H⋯*A*	*D*—H	H⋯*A*	*D*⋯*A*	*D*—H⋯*A*
O3—H31⋯N1^v^	0.81 (2)	2.22 (2)	2.987 (2)	160 (2)
O3—H32⋯N1^vi^	0.81 (2)	2.18 (2)	2.967 (2)	166 (2)

Symmetry codes: (v) 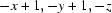; (vi) 
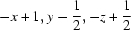
.

**Fig. 1.**
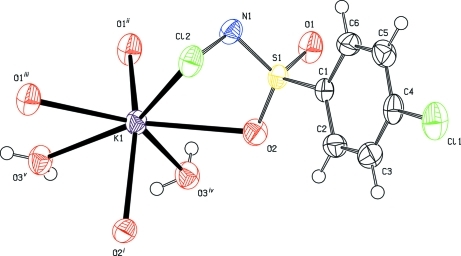
Molecular structure of the title salt hydrate, showing the atom labelling scheme for the asymmetric unit and extended to show the coordination geometry for the K+ cation. The displacement ellipsoids are drawn at the 50% probability level. The H atoms are represented as small spheres of arbitrary radii. See Table 1 for symmetry operations.

**Fig. 2.**
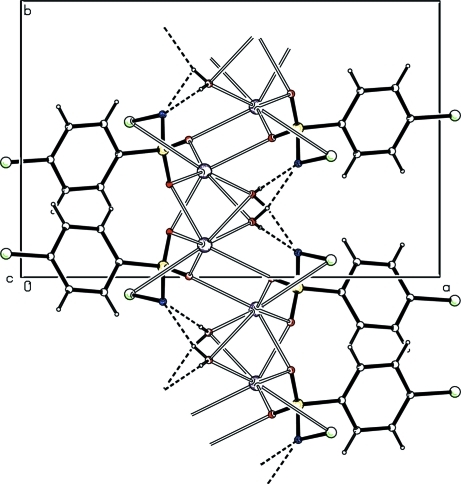
Molecular packing of the title salt hydrate with hydrogen bonding shown as dashed lines.

## supplementary crystallographic information

### Comment

The chemistry of arylsulfonamides and their N-halo compounds is of interest in synthetic, mechanistic, analytical and biological chemistry (Gowda & Shetty, 2004; Usha & Gowda, 2006). To explore the effect of replacing the sodium ion by potassium ion on the solid state structures of *N*-chloroarylsulfonamides (Gowda *et al.*, 2007**a*,b*), in the present work, the structure of potassium *N*-chloro-4-chloro- benzenesulfonamidate monohydrate (I) has been determined (Fig. 1). The structure of (I) resembles those of sodium *N*-chloro-benzenesulfonamide (George *et al.*, 2000) and other sodium *N*-chloro-arylsulfonamides (Olmstead & Power, 1986; Gowda *et al.*, 2007**a*,b*). In particular, there is no interaction between the nitrogen and potassium atom in the molecule.

K^+^ hepta coordination involves two O atoms from bridging water molecules, four sulfonyl O1 atoms from bridging *N*-chloro-4-chlorobenzenesulfonamide anions, and one Cl atom (Table 1). This is in contrast to octahedral coordination of K^+^ in potassium *N*-chloro-benzenesulfonamidate monohydrate by two O atoms from water molecules and four sulfonyl O atoms of four different *N*-chlorobenzenesulfonamide anions (Gowda *et al.*, 2007*a*), and octahedral coordination of Na^+^ in sodium *N*-chloro-4-chlorobenzenesulfonamidate sesquihydrate by three O atoms of water molecules and three sulfonyl O atoms of three different *N*-chloro- 4-chlorobenzenesulfonamide anions (Gowda *et al.*, 2007*b*). The S—N distance of N1—S1, 1.588 (2) Å is consistent with a S—N double bond.

The crystal structure comprises sheets in the *bc* plane which are further stabilized by O—H···N hydrogen bonds (Table 2 and Fig. 2).

### Experimental

The title compound was prepared according to the literature method (Jyothi & Gowda, 2004). The purity of the compound was checked by determining its melting point. Yellow prisms of (I) were obtained from its aqueous solution at room temperature.

### Refinement

The O bound H atoms were located in difference map and later restrained to O—H = 0.82 (2) Å. The other H atoms were positioned with idealized geometry using a riding model with C—H = 0.93 Å. All H atoms were refined with isotropic displacement parameters set to 1.2*U*_eq_ of the parent atom.

### Crystal data

**Table:**

K^+^·C_6_H_4_Cl_2_NO_2_S·H_2_O	*F*(000) = 568
*M**_r_* = 282.18	*D*_x_ = 1.794 Mg m^−^^3^
Monoclinic, *P*2_1_/*c*	Mo *K*α radiation, λ = 0.71073 Å
Hall symbol: -P 2ybc	Cell parameters from 2944 reflections
*a* = 15.487 (1) Å	θ = 3.0–27.7°
*b* = 10.0620 (8) Å	µ = 1.20 mm^−^^1^
*c* = 6.8061 (5) Å	*T* = 293 K
β = 99.888 (7)°	Prism, yellow
*V* = 1044.84 (13) Å^3^	0.42 × 0.42 × 0.30 mm
*Z* = 4	

### Data collection

**Table:**

Oxford Diffraction Xcalibur diffractometer with a Sapphire CCD detector	2139 independent reflections
Radiation source: fine-focus sealed tube	1962 reflections with *I* > 2σ(*I*)
graphite	*R*_int_ = 0.009
Rotation method data acquisition using ω scans	θ_max_ = 26.4°, θ_min_ = 3.4°
Absorption correction: multi-scan (*CrysAlis RED*; Oxford Diffraction, 2009)	*h* = −18→19
*T*_min_ = 0.633, *T*_max_ = 0.715	*k* = −8→12
3796 measured reflections	*l* = −6→8

### Refinement

**Table:**

Refinement on *F*^2^	Secondary atom site location: difference Fourier map
Least-squares matrix: full	Hydrogen site location: inferred from neighbouring sites
*R*[*F*^2^ > 2σ(*F*^2^)] = 0.024	H atoms treated by a mixture of independent and constrained refinement
*wR*(*F*^2^) = 0.067	*w* = 1/[σ^2^(*F*_o_^2^) + (0.0355*P*)^2^ + 0.5896*P*] where *P* = (*F*_o_^2^ + 2*F*_c_^2^)/3
*S* = 1.04	(Δ/σ)_max_ = 0.046
2139 reflections	Δρ_max_ = 0.39 e Å^−^^3^
134 parameters	Δρ_min_ = −0.28 e Å^−^^3^
2 restraints	Extinction correction: *SHELXL97* (Sheldrick, 2008), Fc^*^=kFc[1+0.001xFc^2^λ^3^/sin(2θ)]^-1/4^
Primary atom site location: structure-invariant direct methods	Extinction coefficient: 0.0241 (14)

### Special details

**Table:**

Experimental. CrysAlis RED (Oxford Diffraction, 2009) Empirical absorption correction using spherical harmonics, implemented in SCALE3 ABSPACK scaling algorithm.
Geometry. All e.s.d.'s (except the e.s.d. in the dihedral angle between two l.s. planes) are estimated using the full covariance matrix. The cell e.s.d.'s are taken into account individually in the estimation of e.s.d.'s in distances, angles and torsion angles; correlations between e.s.d.'s in cell parameters are only used when they are defined by crystal symmetry. An approximate (isotropic) treatment of cell e.s.d.'s is used for estimating e.s.d.'s involving l.s. planes.
Refinement. Refinement of *F*^2^ against ALL reflections. The weighted *R*-factor *wR* and goodness of fit *S* are based on *F*^2^, conventional *R*-factors *R* are based on *F*, with *F* set to zero for negative *F*^2^. The threshold expression of *F*^2^ > σ(*F*^2^) is used only for calculating *R*-factors(gt) *etc*. and is not relevant to the choice of reflections for refinement. *R*-factors based on *F*^2^ are statistically about twice as large as those based on *F*, and *R*- factors based on ALL data will be even larger.

### Fractional atomic coordinates and isotropic or equivalent isotropic displacement parameters (Å^2^)

**Table:**

	*x*	*y*	*z*	*U*_iso_*/*U*_eq_	
C1	0.23433 (10)	0.44537 (16)	0.6229 (2)	0.0260 (3)	
C2	0.18971 (12)	0.32599 (18)	0.5954 (3)	0.0342 (4)	
H2	0.2152	0.2528	0.5447	0.041*	
C3	0.10664 (13)	0.3162 (2)	0.6440 (3)	0.0394 (4)	
H3	0.0760	0.2364	0.6266	0.047*	
C4	0.07005 (11)	0.4256 (2)	0.7181 (3)	0.0346 (4)	
C5	0.11456 (13)	0.5446 (2)	0.7494 (3)	0.0383 (4)	
H5	0.0892	0.6170	0.8028	0.046*	
C6	0.19736 (12)	0.55468 (18)	0.7003 (3)	0.0348 (4)	
H6	0.2280	0.6344	0.7193	0.042*	
N1	0.33843 (10)	0.58782 (15)	0.4143 (2)	0.0325 (3)	
O1	0.40091 (8)	0.49624 (15)	0.73235 (19)	0.0411 (3)	
O2	0.35592 (8)	0.33682 (13)	0.4620 (2)	0.0375 (3)	
O3	0.55174 (9)	0.29958 (15)	−0.1344 (2)	0.0401 (3)	
H31	0.5708 (15)	0.324 (2)	−0.231 (3)	0.048*	
H32	0.5890 (13)	0.251 (2)	−0.076 (3)	0.048*	
K1	0.43783 (2)	0.38377 (4)	0.12285 (5)	0.03214 (12)	
Cl1	−0.03614 (3)	0.41509 (7)	0.76911 (8)	0.05237 (17)	
Cl2	0.26012 (3)	0.56182 (5)	0.19737 (7)	0.04134 (14)	
S1	0.33942 (3)	0.46167 (4)	0.55495 (6)	0.02637 (12)	

### Atomic displacement parameters (Å^2^)

**Table:**

	*U*^11^	*U*^22^	*U*^33^	*U*^12^	*U*^13^	*U*^23^
C1	0.0254 (8)	0.0310 (8)	0.0227 (7)	−0.0002 (6)	0.0072 (6)	0.0017 (6)
C2	0.0348 (9)	0.0327 (9)	0.0374 (9)	−0.0024 (7)	0.0129 (7)	−0.0040 (7)
C3	0.0363 (10)	0.0435 (10)	0.0399 (10)	−0.0129 (8)	0.0110 (8)	−0.0030 (8)
C4	0.0251 (8)	0.0527 (11)	0.0274 (8)	−0.0012 (8)	0.0084 (6)	0.0046 (8)
C5	0.0378 (10)	0.0415 (10)	0.0390 (10)	0.0068 (8)	0.0161 (8)	−0.0020 (8)
C6	0.0359 (10)	0.0314 (9)	0.0393 (9)	−0.0032 (7)	0.0131 (8)	−0.0025 (7)
N1	0.0300 (7)	0.0362 (8)	0.0309 (7)	−0.0066 (6)	0.0045 (6)	0.0050 (6)
O1	0.0301 (6)	0.0599 (8)	0.0312 (7)	−0.0064 (6)	−0.0004 (5)	0.0055 (6)
O2	0.0377 (7)	0.0361 (7)	0.0424 (7)	0.0091 (5)	0.0172 (6)	0.0027 (5)
O3	0.0370 (7)	0.0464 (8)	0.0401 (7)	0.0061 (6)	0.0154 (6)	0.0044 (6)
K1	0.0309 (2)	0.0366 (2)	0.0309 (2)	−0.00472 (15)	0.01105 (14)	−0.00100 (15)
Cl1	0.0282 (2)	0.0845 (4)	0.0475 (3)	−0.0038 (2)	0.0153 (2)	0.0031 (3)
Cl2	0.0344 (2)	0.0542 (3)	0.0339 (2)	0.00022 (19)	0.00135 (17)	0.0115 (2)
S1	0.0224 (2)	0.0318 (2)	0.0258 (2)	0.00090 (15)	0.00658 (15)	0.00292 (15)

### Geometric parameters (Å, °)

**Table:**

C1—C6	1.385 (2)	O1—K1^i^	2.7965 (13)
C1—C2	1.382 (2)	O1—K1^ii^	2.8547 (13)
C1—S1	1.7742 (16)	O2—S1	1.4489 (13)
C2—C3	1.386 (3)	O2—K1^iii^	2.6940 (13)
C2—H2	0.9300	O2—K1	2.8576 (13)
C3—C4	1.373 (3)	O3—K1	2.8204 (14)
C3—H3	0.9300	O3—K1^iv^	2.8714 (15)
C4—C5	1.380 (3)	O3—K1^v^	3.1905 (16)
C4—Cl1	1.7414 (18)	O3—H31	0.808 (16)
C5—C6	1.383 (3)	O3—H32	0.808 (16)
C5—H5	0.9300	K1—O2^iv^	2.6940 (13)
C6—H6	0.9300	K1—O1^i^	2.7965 (13)
N1—S1	1.5883 (15)	K1—O1^vi^	2.8547 (13)
N1—Cl2	1.7620 (15)	K1—O3^iii^	2.8714 (15)
N1—K1	3.4014 (16)	K1—O3^v^	3.1905 (16)
O1—S1	1.4460 (13)	K1—Cl2	3.3944 (6)
			
C6—C1—C2	120.77 (15)	O2^iv^—K1—O1^vi^	86.74 (4)
C6—C1—S1	118.90 (13)	O1^i^—K1—O1^vi^	100.42 (3)
C2—C1—S1	120.32 (13)	O3—K1—O1^vi^	65.66 (4)
C3—C2—C1	119.51 (17)	O2^iv^—K1—O2	87.06 (3)
C3—C2—H2	120.2	O1^i^—K1—O2	106.27 (4)
C1—C2—H2	120.2	O3—K1—O2	149.71 (4)
C4—C3—C2	119.30 (17)	O1^vi^—K1—O2	140.52 (4)
C4—C3—H3	120.4	O2^iv^—K1—O3^iii^	84.52 (4)
C2—C3—H3	120.4	O1^i^—K1—O3^iii^	69.60 (4)
C3—C4—C5	121.72 (16)	O3—K1—O3^iii^	77.07 (3)
C3—C4—Cl1	119.22 (15)	O1^vi^—K1—O3^iii^	142.71 (4)
C5—C4—Cl1	119.05 (15)	O2—K1—O3^iii^	75.11 (4)
C4—C5—C6	119.05 (17)	O2^iv^—K1—O3^v^	148.33 (4)
C4—C5—H5	120.5	O1^i^—K1—O3^v^	61.43 (4)
C6—C5—H5	120.5	O3—K1—O3^v^	106.31 (3)
C1—C6—C5	119.64 (17)	O1^vi^—K1—O3^v^	68.17 (4)
C1—C6—H6	120.2	O2—K1—O3^v^	99.95 (4)
C5—C6—H6	120.2	O3^iii^—K1—O3^v^	127.16 (3)
S1—N1—Cl2	108.69 (8)	O2^iv^—K1—Cl2	99.11 (3)
S1—N1—K1	84.87 (6)	O1^i^—K1—Cl2	114.67 (3)
Cl2—N1—K1	74.75 (5)	O3—K1—Cl2	149.42 (3)
S1—O1—K1^i^	144.92 (8)	O1^vi^—K1—Cl2	83.80 (3)
S1—O1—K1^ii^	132.84 (8)	O2—K1—Cl2	58.85 (3)
K1^i^—O1—K1^ii^	79.58 (3)	O3^iii^—K1—Cl2	133.39 (3)
S1—O2—K1^iii^	130.01 (8)	O3^v^—K1—Cl2	60.54 (3)
S1—O2—K1	110.21 (7)	O2^iv^—K1—N1	120.37 (4)
K1^iii^—O2—K1	102.77 (4)	O1^i^—K1—N1	90.00 (4)
K1—O3—K1^iv^	99.34 (4)	O3—K1—N1	159.60 (4)
K1—O3—K1^v^	73.69 (3)	O1^vi^—K1—N1	105.39 (4)
K1^iv^—O3—K1^v^	132.78 (5)	O2—K1—N1	47.20 (4)
K1—O3—H31	140.3 (17)	O3^iii^—K1—N1	110.31 (4)
K1^iv^—O3—H31	90.1 (17)	O3^v^—K1—N1	53.77 (3)
K1^v^—O3—H31	71.8 (17)	Cl2—K1—N1	30.05 (3)
K1—O3—H32	110.2 (17)	N1—Cl2—K1	75.20 (5)
K1^iv^—O3—H32	102.1 (17)	O1—S1—O2	115.65 (8)
K1^v^—O3—H32	124.4 (17)	O1—S1—N1	104.35 (8)
H31—O3—H32	105 (2)	O2—S1—N1	114.45 (8)
O2^iv^—K1—O1^i^	145.96 (4)	O1—S1—C1	107.86 (8)
O2^iv^—K1—O3	78.54 (4)	O2—S1—C1	105.79 (8)
O1^i^—K1—O3	74.49 (4)	N1—S1—C1	108.45 (8)
			
C6—C1—C2—C3	0.7 (3)	Cl2—N1—K1—O1^i^	146.44 (5)
S1—C1—C2—C3	−178.16 (14)	S1—N1—K1—O3	−142.50 (10)
C1—C2—C3—C4	0.2 (3)	Cl2—N1—K1—O3	106.52 (12)
C2—C3—C4—C5	−1.4 (3)	S1—N1—K1—O1^vi^	156.60 (5)
C2—C3—C4—Cl1	177.31 (14)	Cl2—N1—K1—O1^vi^	45.63 (6)
C3—C4—C5—C6	1.6 (3)	S1—N1—K1—O2	9.86 (5)
Cl1—C4—C5—C6	−177.09 (14)	Cl2—N1—K1—O2	−101.12 (6)
C2—C1—C6—C5	−0.5 (3)	S1—N1—K1—O3^iii^	−34.41 (7)
S1—C1—C6—C5	178.40 (14)	Cl2—N1—K1—O3^iii^	−145.38 (5)
C4—C5—C6—C1	−0.6 (3)	S1—N1—K1—O3^v^	−156.23 (8)
K1^iv^—O3—K1—O2^iv^	15.72 (4)	Cl2—N1—K1—O3^v^	92.80 (6)
K1^v^—O3—K1—O2^iv^	147.72 (4)	S1—N1—K1—Cl2	110.97 (8)
K1^iv^—O3—K1—O1^i^	174.75 (5)	S1—N1—Cl2—K1	−79.04 (8)
K1^v^—O3—K1—O1^i^	−53.25 (3)	O2^iv^—K1—Cl2—N1	138.20 (6)
K1^iv^—O3—K1—O1^vi^	−75.88 (5)	O1^i^—K1—Cl2—N1	−37.47 (6)
K1^v^—O3—K1—O1^vi^	56.11 (4)	O3—K1—Cl2—N1	−138.93 (8)
K1^iv^—O3—K1—O2	78.94 (9)	O1^vi^—K1—Cl2—N1	−136.11 (6)
K1^v^—O3—K1—O2	−149.06 (7)	O2—K1—Cl2—N1	57.27 (6)
K1^iv^—O3—K1—O3^iii^	102.66 (7)	O3^iii^—K1—Cl2—N1	47.15 (7)
K1^v^—O3—K1—O3^iii^	−125.35 (3)	O3^v^—K1—Cl2—N1	−67.72 (6)
K1^iv^—O3—K1—O3^v^	−132.00 (5)	K1^i^—O1—S1—O2	85.59 (16)
K1^v^—O3—K1—O3^v^	0.0	K1^ii^—O1—S1—O2	−67.43 (12)
K1^iv^—O3—K1—Cl2	−72.81 (7)	K1^i^—O1—S1—N1	−41.06 (17)
K1^v^—O3—K1—Cl2	59.19 (7)	K1^ii^—O1—S1—N1	165.92 (10)
K1^iv^—O3—K1—N1	−143.50 (10)	K1^i^—O1—S1—C1	−156.25 (14)
K1^v^—O3—K1—N1	−11.50 (12)	K1^ii^—O1—S1—C1	50.73 (13)
S1—O2—K1—O2^iv^	−149.02 (5)	K1^iii^—O2—S1—O1	26.81 (12)
K1^iii^—O2—K1—O2^iv^	69.17 (7)	K1—O2—S1—O1	−101.25 (8)
S1—O2—K1—O1^i^	62.84 (8)	K1^iii^—O2—S1—N1	148.17 (8)
K1^iii^—O2—K1—O1^i^	−78.97 (5)	K1—O2—S1—N1	20.11 (10)
S1—O2—K1—O3	149.81 (7)	K1^iii^—O2—S1—C1	−92.49 (10)
K1^iii^—O2—K1—O3	8.00 (11)	K1—O2—S1—C1	139.44 (7)
S1—O2—K1—O1^vi^	−67.75 (10)	Cl2—N1—S1—O1	−176.41 (8)
K1^iii^—O2—K1—O1^vi^	150.44 (5)	K1—N1—S1—O1	111.60 (6)
S1—O2—K1—O3^iii^	125.88 (8)	Cl2—N1—S1—O2	56.20 (11)
K1^iii^—O2—K1—O3^iii^	−15.93 (4)	K1—N1—S1—O2	−15.79 (8)
S1—O2—K1—O3^v^	−0.13 (8)	Cl2—N1—S1—C1	−61.63 (10)
K1^iii^—O2—K1—O3^v^	−141.94 (4)	K1—N1—S1—C1	−133.62 (6)
S1—O2—K1—Cl2	−46.53 (6)	C6—C1—S1—O1	61.22 (16)
K1^iii^—O2—K1—Cl2	171.66 (5)	C2—C1—S1—O1	−119.88 (15)
S1—O2—K1—N1	−11.49 (6)	C6—C1—S1—O2	−174.45 (14)
K1^iii^—O2—K1—N1	−153.30 (7)	C2—C1—S1—O2	4.44 (16)
S1—N1—K1—O2^iv^	61.26 (7)	C6—C1—S1—N1	−51.24 (16)
Cl2—N1—K1—O2^iv^	−49.71 (6)	C2—C1—S1—N1	127.66 (14)
S1—N1—K1—O1^i^	−102.59 (6)		

Symmetry codes: (i) −*x*+1, −*y*+1, −*z*+1; (ii) *x*, *y*, *z*+1; (iii) *x*, −*y*+1/2, *z*+1/2; (iv) *x*, −*y*+1/2, *z*−1/2; (v) −*x*+1, −*y*+1, −*z*; (vi) *x*, *y*, *z*−1.

### Hydrogen-bond geometry (Å, °)

**Table:**

*D*—H···*A*	*D*—H	H···*A*	*D*···*A*	*D*—H···*A*
O3—H31···N1^v^	0.81 (2)	2.22 (2)	2.987 (2)	160 (2)
O3—H32···N1^vii^	0.81 (2)	2.18 (2)	2.967 (2)	166 (2)

Symmetry codes: (v) −*x*+1, −*y*+1, −*z*; (vii) −*x*+1, *y*−1/2, −*z*+1/2.
